# Serum urate and cardiovascular events in the DCCT/EDIC study

**DOI:** 10.1038/s41598-021-90785-4

**Published:** 2021-07-09

**Authors:** Alicia J. Jenkins, Barbara H. Braffett, Arpita Basu, Ionut Bebu, Samuel Dagogo-Jack, Trevor J. Orchard, Amisha Wallia, Maria F. Lopes-Virella, W. Timothy Garvey, John M. Lachin, Timothy J. Lyons, the DCCT/EDIC Research Group, D. M. Nathan, B. Zinman, D. M. Nathan, J. Lachin, I. Bebu, B. Braffett, J. Backlund, L. Diminick, L. El Ghormli, X. Gao, D. Kenny, K. Klumpp, M. Lin, V. Trapani, E. Leschek, M. Steffes, A. Karger, J. Seegmiller, V. Arends, Y. Pokharel, M. Barr, C. Campbell, S. Hensley, J. Hu, L. Keasler, Y. Li, T. Taylor, Z. M. Zhang, B. Blodi, R. Danis, D. Lawrence, H. Wabers, A. Jacobson, C. Ryan, D. Saporito

**Affiliations:** 1 Division of Endocrinology, Clinical Sciences Building, Suite 822, Medical University of South Carolina, 96 Jonathan Lucas Street, Charleston, SC 29425 USA; 2 National Health and Medical Research Council Clinical Trials Centre, University of Sydney, Sydney, Australia; 3 George Washington University Biostatistics Center, Rockville, MD USA; 4 University of Nevada, Las Vegas, NV USA; 5 University of Tennessee Health Science Center, Memphis, TN USA; 6 University of Pittsburgh, Pittsburgh, PA USA; 7 Northwestern University, Chicago, IL USA; 8 Ralph H. Johnson Veterans Affairs Medical Center, Charleston, SC USA; 9 University of Alabama at Birmingham, Birmingham VA Medical Center, Birmingham, AL USA; 10 Diabetes Free SC, BlueCross BlueShield of South Carolina, Columbia, SC USA; 11 Massachusetts General Hospital, Boston, USA; 12 University of Toronto, Toronto, Canada; 13 Vanderbilt University, Nashville, USA; 14 Case Western Reserve University, Cleveland, USA; 15 Weill Cornell Medical College, New York, USA; 16 Henry Ford Health System, Detroit, USA; 17 International Diabetes Center, Minneapolis, USA; 18 Joslin Diabetes Center, Boston, USA; 19 Mayo Clinic, Rochester, USA; 20 Medical University of South Carolina, Charleston, USA; 21 Northwestern University, Evanston, USA; 22 University of California, San Diego, USA; 23 University of Iowa, Iowa, USA; 24 University of Maryland, Baltimore, USA; 25 University of Michigan, Ann Arbor, USA; 26 University of Minnesota, Minneapolis, USA; 27 University of Missouri, Columbia, USA; 28 University of New Mexico, Albuquerque, USA; 29 University of Pennsylvania, Philadelphia, USA; 30 University of Pittsburgh, Pittsburgh, USA; 31 University of South Florida, Tampa, USA; 32 University of Tennessee, Memphis, USA; 33 University of Texas, Austin, USA; 34 University of Washington, Seattle, USA; 35 University of Western Ontario, London, Canada; 36 Vanderbilt University, Nashville, USA; 37 Washington University, St. Louis, USA; 38 Yale University, New Haven, USA; 39 Albert Einstein College of Medicine, Bronx, USA; 40 The Biostatistics Center, George Washington University, Bethesda, USA; 41 National Institute of Diabetes, Digestive and Kidney Diseases, National Institutes of Health, Bethesda, USA; 42 Central Biochemistry Laboratory (University of Minnesota), Minneapolis, USA; 43 Central ECG Reading Unit (Wake Forest School of Medicine), Winston-Salem, USA; 44 Central Ophthalmologic Reading Unit (University of Wisconsin), Madison, USA; 45 Central Neuropsychological Reading Unit (NYU Winthrop Hospital, University of Pittsburgh), Pittsburgh, USA

**Keywords:** Endocrinology, Risk factors

## Abstract

In type 2 diabetes, hyperuricemia is associated with cardiovascular disease (CVD) and the metabolic syndrome (MetS), but associations in type 1 diabetes (T1D) have not been well-defined. This study examined the relationships between serum urate (SU) concentrations, clinical and biochemical factors, and subsequent cardiovascular events in a well-characterized cohort of adults with T1D. In 973 participants with T1D in the Diabetes Control and Complications Trial/Epidemiology of Diabetes Interventions and Complications Study (DCCT/EDIC), associations were defined between SU, measured once in blood collected 1997–2000, and (a) concurrent MetS and (b) incident ‘any CVD’ and major adverse cardiovascular events (MACE) through 2013. SU was higher in men than women [mean (SD): 4.47 (0.99) vs. 3.39 (0.97) mg/dl, respectively, p < 0.0001], and was associated with MetS features in both (men: p = 0.0016; women: p < 0.0001). During follow-up, 110 participants (11%) experienced “any CVD”, and 53 (5%) a MACE. Analyzed by quartiles, SU was not associated with subsequent CVD or MACE. In women, SU as a continuous variable was associated with MACE (unadjusted HR: 1.52; 95% CI 1.07–2.16; p = 0.0211) even after adjustment for age and HbA1c (HR: 1.47; 95% CI 1.01–2.14; p = 0.0467). Predominantly normal range serum urate concentrations in T1D were higher in men than women and were associated with features of the MetS. In some analyses of women only, SU was associated with subsequent MACE. Routine measurement of SU to assess cardiovascular risk in T1D is not merited.

**Trial registration** clinicaltrials.gov NCT00360815 and NCT00360893.

## Introduction

Elevated serum concentrations of urate (SU) may be caused by inherited disorders of metabolism and/or acquired conditions, such as the metabolic syndrome (MetS), renal impairment, obesity, and type 2 diabetes (T2D)^1,2^. The MetS can also occur in type 1 diabetes (T1D) and is associated with increased risk for micro- and macrovascular complications^3,4^. Regardless of cause, hyperuricemia has been implicated in gout, cardiovascular disease (CVD), hypertension, and renal injury^5,6^. SU concentrations are higher in men than women, yet for reasons not fully elucidated, associations with macrovascular disease are stronger in women^7^. The sex-specific associations between SU and subsequent vascular events in T1D remain poorly defined. To address this deficit, we utilized the remarkable long-term data collected by the Diabetes Control and Complications Trial/Epidemiology of Diabetes Interventions and Complications (DCCT/EDIC) study.

For SU determination, we used samples from DCCT/EDIC participants who had no clinically evident macrovascular disease at “baseline”, defined for this purpose as 1997–2000. We determined sex-specific associations of SU with concurrent clinical status including features of the MetS^4,8–11^, then related SU to ‘any CVD’ event and ‘major adverse cardiovascular events’ (MACE) occurring over a median of 14 years of follow-up. Due to known sex differences in SU concentrations and associations with CVD^7^, and of sex differences in risk factors such as CRP, insulin sensitivity and lipoproteins^12–14^, sex-specific analyses were performed.

### Subjects and study design

The study met the Declaration of Helsinki guidelines and was approved by the MUSC Institutional Review Board and all participating DCCT/EDIC centers. Each participant was over 18 years of age and provided written informed consent. The design, methods, and outcomes of the DCCT/EDIC study have been detailed previously^15,16^. Briefly, the DCCT aimed to determine if intensive diabetes management could prevent or delay the onset and/or progression of microvascular complications in T1D. Between 1983 and 1989, 1441 participants with T1D aged 13–39 years were randomized to conventional (n = 730) or intensive (n = 711) diabetes therapy^16^. In parallel, two cohorts were defined: a primary prevention cohort (n = 726) with 1–5 years duration of T1D, no retinopathy and urinary albumin excretion rate (AER) < 40 mg/24 h; and a secondary intervention cohort (n = 715) with 1–15 years duration of T1D, mild-to-moderate non-proliferative retinopathy and urinary AER < 200 mg/24 h. Baseline exclusion criteria included hypertension (≥ 140/90 mmHg or medication use), hyperlipidemia (fasting serum cholesterol level ≥ 3 SD above age- and sex-specific means or medication use), other CVD, and other significant medical conditions. In 1993, after an average of 6.5 years follow-up, the DCCT was terminated early because of clear-cut benefit of intensive treatment on the primary end-point, retinopathy, and similar benefits on nephropathy and neuropathy^16^. In 1994, the majority (96%) of the surviving cohort entered the observational follow-up EDIC study^15^, which continues today with 94% of survivors continuing participation. During DCCT, the mean HbA1c in the intensive vs. conventional treatment arms were 7.2% and 9.1%, respectively, but throughout EDIC the two treatment arms have maintained similar HbA1c of 8.0%^15^.

In 1996, a collaboration between the DCCT/EDIC and the Medical University of South Carolina (MUSC) was established to identify markers and mechanisms for vascular complications of diabetes, and 25 of 28 DCCT/EDIC clinical centers participated. Among 987 participants, 973 (98.6%) (540 men, 433 women) were free of CVD at the time of blood collection for the present study (1997–2000). These subjects represented 71% of the 1375 EDIC participants and were representative of the entire DCCT/EDIC cohort.

## Methods

### Sample collection, clinical measurements and definitions

During annual EDIC assessments, height, weight, blood pressure, and pulse rate were recorded, and venous blood was collected and sent to the DCCT/EDIC Central Biochemistry Laboratory (CBL, University of Minnesota) for measurement of HbA1c by high performance liquid chromatography. Fasting lipids (triglycerides, total- and HDL-cholesterol) and AER were measured in alternate years. Fasting blood was also drawn for the MUSC study and serum aliquots were prepared and shipped overnight on ice to MUSC, then stored (− 80 °C) until thawed for SU and other research measures. LDL-cholesterol was calculated using the Friedewald equation. Kidney disease was defined as microalbuminuria (i.e. AER ≥ 30 mg/24 on ≥ 2 consecutive visits) during DCCT/EDIC. Estimated glomerular filtration rates (eGFR) were calculated from serum creatinine measured annually during EDIC. A measure of insulin sensitivity, the estimated glucose disposal rate (eGDR, a function of waist-to hip ratio, history of hypertension, and HbA1c) was calculated using an equation developed by Williams et al*.*^17^. For the present study, SU was measured on a single occasion in fasting serum samples collected 1997–2000.

Hyperlipidemia was defined as LDL-C ≥ 130 mg/dl or use of lipid-lowering medications. Hypertension was defined as one or more of: systolic blood pressure ≥ 140 mmHg, diastolic blood pressure ≥ 90 mmHg, documented hypertension, or use of anti-hypertensive medications, including angiotensin converting enzyme (ACE) inhibitors or angiotensin II receptor blocker (ARB) drugs for any reason. Metabolic syndrome (MetS) was defined as having two or more of the following during EDIC: (1) systolic BP ≥ 130 mmHg, diastolic ≥ 85 mm Hg, or use of anti-hypertensive medications for any reason; (2) waist circumference ≥ 88 cm (women) or ≥ 102 cm (men); (3) HDL-cholesterol < 50 mg/dl (women) or < 40 mg/dl (men); and (4) fasting triglycerides ≥ 150 mg/dl.

CVD events were identified through 2013 and were adjudicated by a committee masked to DCCT treatment assignment and HbA1c. “Any CVD” was defined as any of the following: non-fatal myocardial infarction (MI); stroke; death judged to be secondary to CVD; subclinical (“silent”) MI detected on an annual electrocardiogram; angina confirmed by ischemic changes with exercise tolerance testing or clinically significant obstruction on coronary angiography; congestive heart failure (with paroxysmal nocturnal dyspnea, orthopnea, marked limitation of physical activity caused by heart disease); angioplasty and/or coronary artery bypass. MACE was defined as any of the following: CVD death, non-fatal MI, or stroke^18^.

### Research biochemistry

Serum urate was measured on first-thaw sera by an enzymatic/spectrophotometric assay detecting hydrogen peroxide using an auto-analyzer at the MUSC Central Laboratory^19^. The normal reference range of SU is 1.5–6.0 mg/dl in women and 2.5–7.0 mg/dl in men. Elevated concentrations are defined as being above these upper limits^7^, or alternately, a combined level of > 6.8 mg/dl^2^. In the present work, sex-specific SU concentrations are presented as quartiles and as continuous variables.

C-Reactive Protein (CRP) was determined in sera by high-sensitivity nephelometry (Nephelometer 100, Dade Behring, Marburg, Germany) as previously described, with the lower limit of detection being 0.2 mg/l, and intra- and inter-assay CVs < 2% and < 11%, respectively^12^.

### Statistical analyses

Analyses were performed in SAS 9.4 (SAS Institute Inc., Cary, North Carolina). Participant characteristics at the time of sample acquisition (1997–2000) were reported by sex-specific SU quartiles. Comparisons between quartiles were made using the Kruskal–Wallis test for continuous variables and chi-square test for categorical variables. Cox proportional hazards regression models were used to evaluate the relationship between SU, as a fixed covariate, and subsequent “any CVD” and MACE (as defined in “Methods”), separately by sex. Serum urate, both as a continuous variable and by quartiles, was evaluated for each sex as a predictor of vascular outcomes. Models are presented unadjusted and minimally adjusted for concurrent age and mean DCCT/EDIC HbA1c, defined as the cumulative exposure from DCCT baseline to the time of the SU measurement, with weights of 0.25 and 1.0 assigned to quarterly DCCT and annual EDIC values, respectively. Statistical significance was defined as p < 0.05. Adjustments for multiple comparisons were not made due to limited statistical power.

### Ethics approval and consent to participate

Ethical approval was obtained by the institutional review boards at all sites and all participants provided written informed consent to participate in the study.

## Results

### Distribution of serum urate concentrations

**Figure 1:**
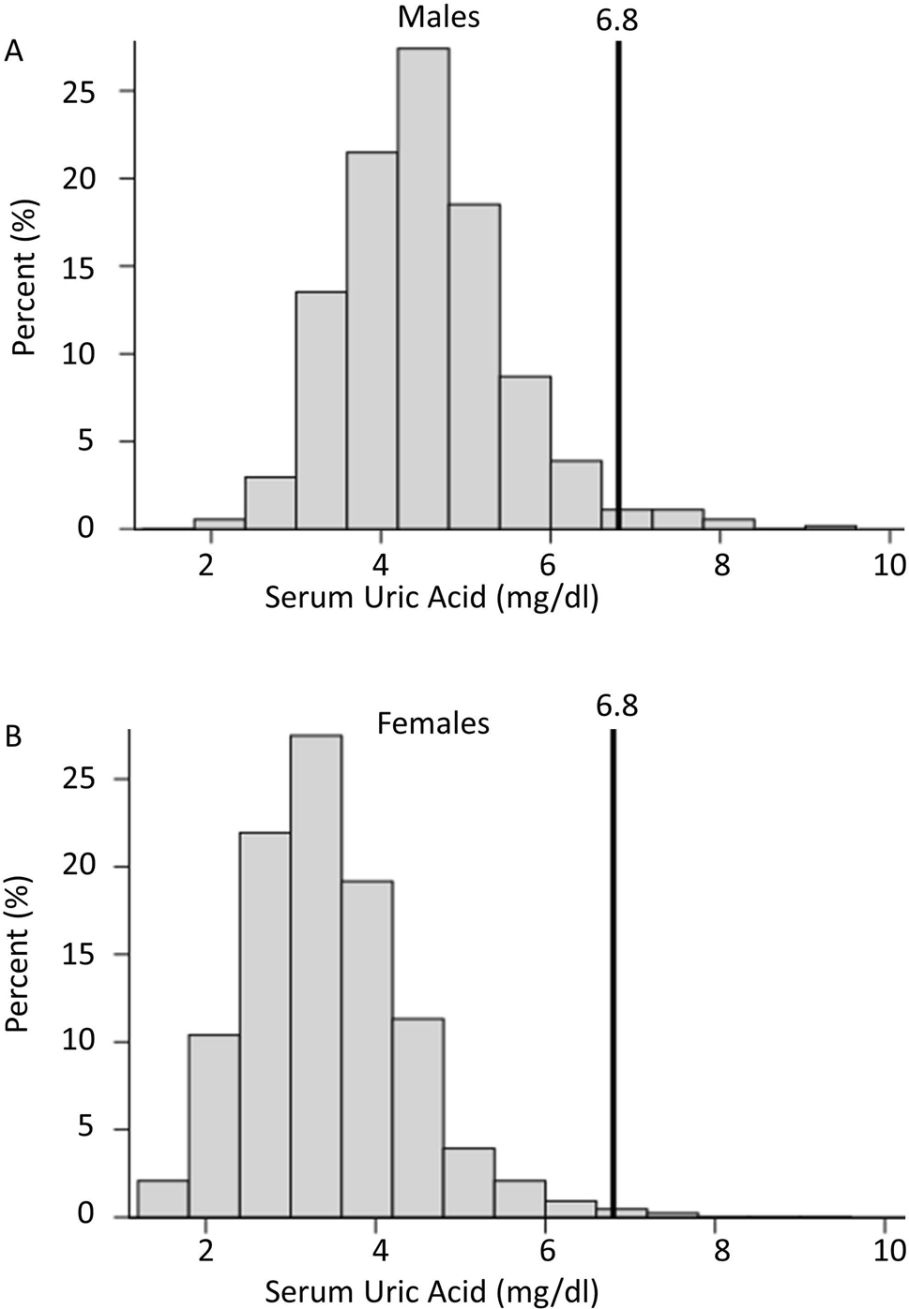
Distribution of serum urate concentrations in (**A**) 540 men and (**B**) 433 women with type 1 diabetes from the DCCT/EDIC cohort in 1997–2000. The vertical lines indicate the upper limit of normal range, 6.8 mg/dl.

As shown in Fig. 1, SU was higher in men than women (p < 0.0001). It was normally distributed in both sexes, with only 1.4% of adults having elevated concentrations (> 6.8 mg/dl)^2^, or if sex-specific cut-points were employed, 1.6% of women (> 6.0 mg/dl) and 2.0% of men (> 7.0 mg/dl)^7^.

### Characteristics of participants by serum urate quartile

Demographic, clinical, and biochemical characteristics for men and women according to serum urate quartiles are shown in Table 1. In both sexes, higher SU was associated with higher BMI, higher mean DCCT/EDIC HbA1c, higher triglycerides, lower eGFR, the presence of microalbuminuria, and the presence of MetS. For men only, higher SU was associated with smoking, DCCT conventional treatment group, and systolic blood pressure. For women only, higher SU was associated with younger age, DCCT secondary intervention cohort, longer duration of diabetes, higher pulse rate, hypertension, higher total and LDL-cholesterol concentrations, lower HDL-cholesterol concentrations, lower eGDR, higher CRP, and use of ACE or ARB medications.

**Table 1:** EDIC participant characteristics by quartiles of SU in men and women (at time of SU sampling, 1997–2000).

Serum urate (mg/dl)	Men (n = 540)				p*	Women (n = 433)				p*
	1.8–3.8 n = 140	3.9–4.3 n = 131	4.4–5.0 n = 140	5.1–9.2 n = 129		1.3–2.7 n = 112	2.8–3.2 n = 100	3.3–3.9 n = 116	4.0–7.7 n = 105	
Age (years)	40.3 (6.4)	40.1 (6.8)	39.9 (6.8)	39.9 (6.9)	0.9959	40.2 (6.3)	38.5 (7.3)	39.8 (7.2)	37.7 (7.8)	**0.0370**
**Design**										
Treatment group (% intensive)	57.1	54.2	50.0	40.3	**0.0357**	52.7	49.0	58.6	48.6	0.4099
Cohort (% primary prevention)	53.6	43.5	52.1	50.4	0.3639	50.9	65.0	46.6	42.9	**0.0091**
**Behavioral**										
BMI (kg/m^2^)	26.3 (3.4)	26.6 (3.3)	27.7 (4.2)	27.8 (4.2)	**0.0024**	24.8 (3.4)	26.2 (4.0)	27.0 (4.6)	27.5 (4.7)	**< 0.0001**
Cigarette smoker (%)	24.3	22.1	16.4	12.4	0.0536	20.5	17.0	19.8	17.1	0.8726
**Clinical**										
Diabetes duration (years)	17.1 (4.9)	17.7 (4.8)	17.2 (4.7)	17.2 (4.4)	0.6407	17.7 (4.8)	16.4 (4.6)	18.1 (5.0)	18.3 (4.8)	**0.0095**
Serum urate (mg/dl)	3.4 (0.4)	4.1 (0.1)	4.7 (0.2)	5.8 (0.7)	–-	2.3 (0.4)	3.0 (012)	3.6 (0.2)	4.7 (0.7)	–-
Pulse rate (bpm)	69 (11)	68 (12)	68 (12)	71 (11)	0.1610	73 (9)	73 (11)	75 (12)	77 (11)	**0.0040**
Blood pressure (mm Hg)										
Systolic	122 (14)	121 (13)	122 (11)	126 (15)	**0.0294**	114 (12)	115 (13)	117 (15)	118 (16)	0.2755
Diastolic	77 (10)	77 (9)	77 (9)	79 (9)	0.0723	72 (8)	72 (9)	73 (9)	74 (9)	0.4813
Hypertension (%)	24.3	25.2	27.9	34.9	0.2116	10.7	15.0	21.6	32.4	**0.0005**
HbA1c (%)	8.2 (1.2)	8.2 (1.4)	8.2 (1.2)	8.2 (1.4)	0.9981	8.3 (1.3)	8.2 (1.2)	8.2 (1.5)	8.2 (1.5)	0.9356
DCCT/EDIC HbA1c (%)	8.0 (1.1)	8.1 (1.2)	8.1 (1.1)	8.4 (1.2)	**0.0273**	8.0 (19.0)	8.1 (1.1)	8.2 (1.2)	8.5 (1.4)	**0.0254**
Fasting lipids (mg/dl)										
Total cholesterol	185 (34)	186 (30)	191 (36)	195 (41)	0.0906	181 (30)	186 (33)	191 (32)	195 (38)	**0.0466**
Triglycerides	86 ( 47)	87 (58)	100 (85)	119 (92)	**0.0003**	65 (27)	69 (39)	82 (53)	96 (58)	**< 0.0001**
LDL-cholesterol	116 (30)	117 (28)	119 (31)	120 (34)	0.6632	103 (27)	108 (30)	112 (30)	117 (30)	**0.0091**
HDL-cholesterol	52 (12)	51 (11)	51 (13)	52 (14)	0.7087	65 (15)	64 (13)	63 (14)	58 (15)	**0.0141**
Hyperlipidemia (%)	32.9	34.4	37.9	36.4	0.8281	17.0	21.0	29.3	40.0	**0.0007**
Insulin dose (units/kg/day)	0.67 (0.2)	0.67 (0.2)	0.65 (0.2)	0.70 (0.2)	0.0937	0.59 (0.2)	0.62 (0.2)	0.65 (0.2)	0.66 (0.2)	**0.0300**
Estimated GFR(ml/min/1.73m^2^)	113 (11)	111 (14)	108 (12)	104 (19)	**0.0004**	111 (13)	111 (13)	108 (14)	101 (24)	**0.0068**
Sustained AER ≥ 30 (%)	13.8	13.2	15.9	29.6	**0.0014**	2.8	6.1	9.7	17.4	**0.0021**
Metabolic syndrome (%)^a^	15.0	13.0	25.0	29.5	**0.0016**	6.3	8.0	18.1	35.2	**< 0.0001**
Estimated GDR (mg/kg/min)	8.0 (1.8)	8.0 (1.9)	7.7 (2.0)	7.6 (2.3)	0.2372	10.0 (1.4)	9.9 (1.7)	9.4 (2.0)	8.9 (2.2)	**0.0005**
CRP (mg/l)^b^	0.30 (0.4)	0.40 (1.1)	0.33 (0.6)	0.42 (0.6)	0.7100	0.54 (0.9)	0.76 (1.2)	0.90 (1.2)	0.95 (1.7)	**0.0048**
**Medications**										
Lipid-lowering (%)	5.7	9.9	9.3	10.1	0.5347	3.6	3.0	3.5	8.6	0.1749
ACE/ARB (%)	15.0	16.8	18.6	21.7	0.5286	5.4	6.0	7.8	23.8	**< 0.0001**
Aspirin (%)	10.7	16.0	10.7	12.4	0.5085	5.4	10.0	6.9	7.6	0.6323

Data are means (SD) or %. Quartile groups are not equal in sample size due to the discrete nature of the data and the presence of ties.

*SU* serum urate, *BMI* body mass index, *LDL* low-density lipoprotein, *HDL* high-density lipoprotein, *GFR* glomerular filtration rate, *AER* albumin excretion rate, *GDR* glucose disposal rate, *CRP* C-reactive protein, *ACE* angiotensin-converting enzyme, *ARB* angiotensin-receptor blocker.

*p-values from the Kruskal–Wallis test for continuous variables and the χ^2^ test for categorical variables.

^a^Metabolic syndrome is defined as having two or more of the following four during EDIC: (1) SBP ≥ 130 mmHg or DBP ≥ 85 mm Hg or any anti-hypertensive medication including ACE/ARB for any reason; (2) female waist ≥ 88 cm or male waist ≥ 102 cm; (3) female HDL < 50 mg/dl or male HDL < 40 mg/dl; (4) triglycerides ≥ 150 mg/dl.

^b^CRP was available in 434 men and 341 women.

### Association between serum urate and MetS and eGDR

The prevalence of MetS at the time of SU sampling did not differ significantly between prior DCCT randomization groups (Intensive 19.2% vs. Conventional 18.6%; p = 0.8126) but was higher in the secondary intervention than the primary prevention group (22.8% vs. 15.1% respectively, p < 0.0020). SU did not differ according to these prior DCCT categories. As shown in Table 1, the percentage with MetS increased significantly across SU quartiles for both men (twofold, p = 0.002) and women (by > fivefold, p < 0.0001). SU concentrations were higher in men with (n = 111) than without (n = 429) the MetS (4.8 ± 1.2 vs. 4.4 ± 0.9 mg/dl, respectively, p = 0.0003), and also in women with (n = 73) than without (n = 360) the MetS (4.0 ± 1.1 vs. 3.3 ± 0.9 mg/dl, respectively, p < 0.0001). SU (as a continuous variable) and eGDR, a measure of insulin sensitivity, were inversely correlated with SU in both men (r = − 0.146, p = 0.0007) and women (r = − 0.269, p < 0.0001).

### Associations between serum urate and CRP concentrations

CRP levels were higher in women than men [0.788 (1.24) and 0.359 (0.716) mg/l, respectively, p < 0.0001]. In addition to the association between SU quartiles and CRP in women, mean (SD) SU also correlated with concurrent CRP in women (r = 0.155, p = 0.0041), but not in men (r = 0.092, p = 0.0568). The statistically significant SU-CRP correlation in women did not persist after adjustment for age, BMI and HbA1c (p = 0.0939).

Using the same CRP-defined CVD risk categories for both sexes, (low risk CRP: < 1 mg/l; medium risk: 1–3 mg/l; high risk: > 3 mg/l)^20^, respective serum urate concentrations (mg/dl, mean ± SD) in women were: 3.4 ± 1.0 (n = 357), 3.6 ± 1.0 (n = 58), and 3.5 ± 1.0 (n = 18) mg/dl, p = 0.1325; and in men: 4.4 ± 0.9 (n = 509), 5.1 ± 1.3 (n = 26), and 5.0 ± 1.8 (n = 5), p = 0.0186. Thus, in men only, CRP-defined CVD risk category was associated with significantly higher SU.

### Risk of future CVD/MACE according to serum urate

There were 110 participants (11%) who experienced “any CVD” event, including 61 men (11%) and 49 women (11%). In the combined cohort (both sexes), there were no significant associations between baseline serum urate as a continuous variable and subsequent “any CVD” events (HR = 1.15; 95% CI 0.97, 1.35) or MACE (HR = 1.22; 95% CI 0.97, 1.54). This unstratified model assumes that the background cardiovascular risk is the same for the men and women. A sex-stratified model, allowing for the possibility that background risk differs, yielded similar results (HR = 1.19; 95% CI 1.00, 1.43 for “any CVD”, and HR = 1.30; 95% CI 1.01, 1.67 for MACE).

Sex-specific associations between baseline SU and subsequent CVD during EDIC are presented in Table 2. Three women had more than one event (MI followed by CVD death). In women, but not in men, SU was significantly associated with subsequent MACE in both unadjusted (p = 0.0211) and minimally adjusted (p = 0.0466) models. In models further adjusted separately for MetS, triglycerides, or eGFR, the relationship between SU and MACE in women persisted (p = 0.0447, p = 0.0417, and p = 0.0187 respectively, data not shown); however, the effect was fully attenuated with adjustment for microalbuminuria (p = 0.3582). Elimination from the analyses of the 12 participants (6 men, 6 women) whose GFR was < 60 ml/min/1.73 m^2^ at the time of SU measurement did not materially alter these findings.

**Table 2:** Association between SU as a continuous variable and subsequent CVD and MACE events by sex, unadjusted and with minimal adjustment for age and mean DCCT/EDIC HbA1c.

	Men (n = 540)			Women (n = 433)		
	No. of Participants (%)^a^	HR (95% CI)^b^ p-value unadjusted	HR 95% CI)^b^ p-value adjusted	No. of Participants (%)^a^	HR (95% CI)^b^ p-value unadjusted	HR (95% CI)^b^ p-value adjusted
**Any cardiovascular disease event**	61 (11)	1.18 (0.92–1.50) 0.1920	1.15 (0.90–1.46) 0.2717	49 (11)	1.22 (0.93–1.60) 0.1544	1.17 (0.88–1.56) 0.2833
1. Non-fatal acute myocardial infarction	16 (3)			15 (3)		
2. Non-fatal cerebrovascular event	7 (1)			7 (2)		
3. Death from cardiovascular disease	6 (1)			5 (1)		
4. Silent myocardial infarction	13 (2)			14 (3)		
5. Confirmed angina	6 (1)			12 (3)		
6. Revascularization	37 (7)			22 (5)		
7. Congestive heart failure	3 (1)			1 (< 1)		
**Non-fatal MI or stroke or death from cardiovascular disease (MACE)**	29 (5)	1.13 (0.79–1.61) 0.5083	1.10 (0.77–1.58) 0.5944	24 (6)	1.52 (1.07–2.16) **0.0211**	1.47 (1.01–2.14) **0.0466**
1. Non-fatal acute myocardial infarction	16 (3)			15 (3)		
2. Non-fatal cerebrovascular event	7 (1)			7 (2)		
3. Death from cardiovascular disease	6 (1)			5 (1)		

^a^Number of participants with each type of event, regardless of whether or not it is the initial event for that subject (including recurrent events).

^b^Cox proportional hazard regression models minimally adjusted for age and mean DCCT/EDIC HbA1c as fixed covariates at the time of the SU measurement.

**Figure 2:**
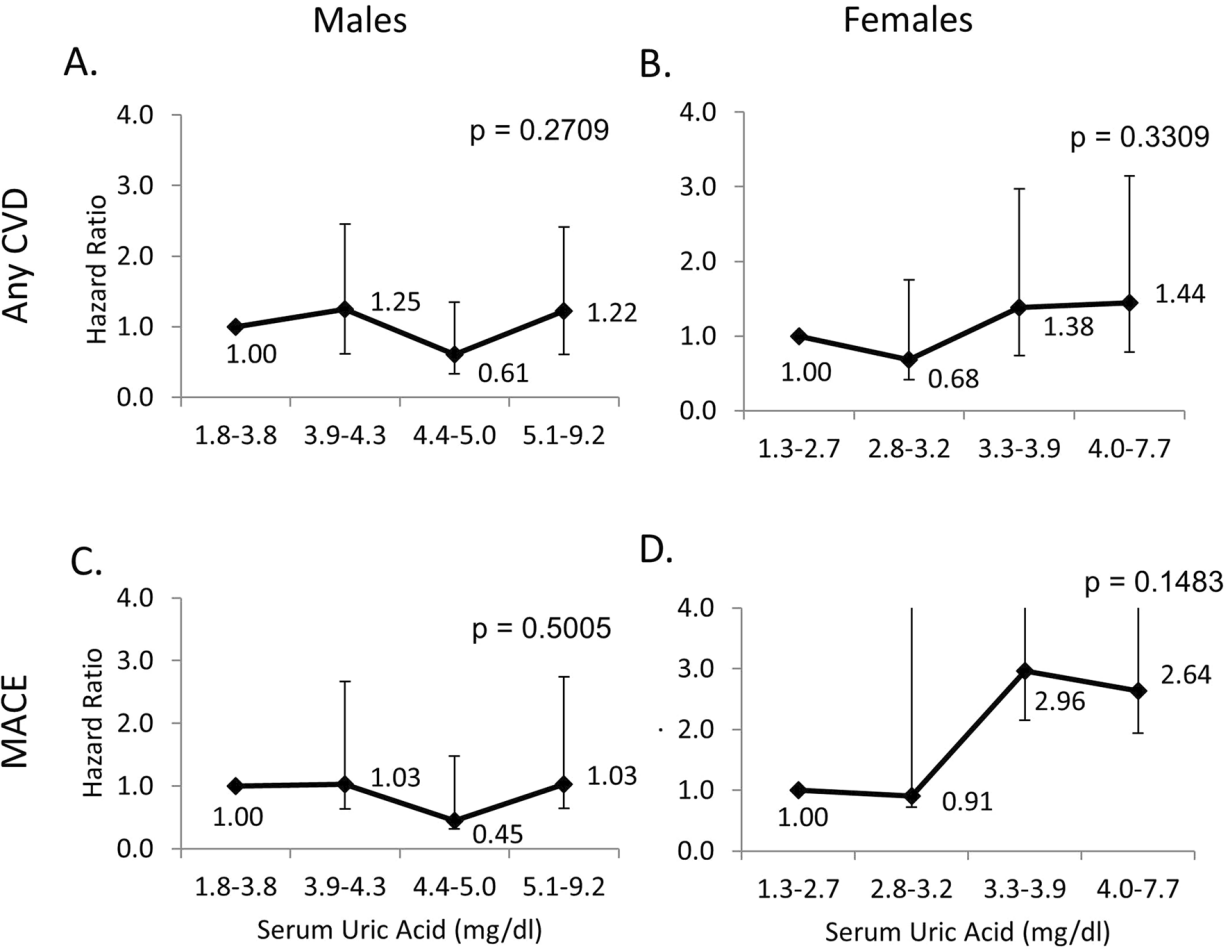
Hazard ratios for risk of ‘any CVD’ and MACE in men and women with type 1 diabetes, according to serum urate quartiles (relative to the lowest quartile). Hazard ratios for ‘any CVD’ are shown in (**A**) for men and (**B**) for women; those for MACE are shown in (**C**) for men and (**D**) for women.

There were no significant associations between SU quartiles and subsequent “any CVD” events or MACE for either sex (Fig. 2). As only 14 subjects had elevated SU concentrations, we did not attempt to analyze CVD events according to presence or absence of elevated SU concentrations.

## Discussion

In this study of middle-aged and older adults with T1D from the DCCT/EDIC cohort, we demonstrated generally normal serum urate concentrations, with higher values in men than women, as expected. SU concentrations were associated with features of the MetS, especially in women. In a longitudinal analysis through 2013, ‘baseline’ SU concentrations (as a continuous variable) were associated with subsequent MACE in women only, no association was found with “any CVD” in either sex. Results do not support the routine measurement of urate in T1D for CVD risk assessment.

### Cross-sectional associations between urate and vascular risk factors

Whilst SU levels were relatively normal in our DCCT/EDIC sub-study, we observed correlations with hypertension, RAAS drugs, features of MetS, and renal dysfunction. SU was associated with hypertension and use of ACE/ARB drugs in women, but not in men. In cross-sectional studies by others, higher urate concentrations have been associated with hypertension, increased arterial stiffness and RAAS activation^21^. Relationships of urate with ACE/ARB medication may be explained by use of these agents in people with increased albuminuria and/or with recognized CVD risk, and by the association of renal dysfunction with elevated urate concentrations^1,2^ We also noted higher SU concentrations in those with than without MetS, and in both sexes, SU concentrations increased with the number of features of MetS present. Higher SU concentrations were associated with concurrent renal dysfunction, reflected by both lower eGFR and sustained increases in AER (Table 1).

In T1D, links between the MetS and increased risk of renal/cardiovascular disease, and between renal dysfunction and cardiovascular disease or death are well recognized^22–24^. Two prior studies explored cross-sectional relationships between urate concentrations and CVD in T1D. In one, a study of 3800 adults with T1D, high SU concentrations (> 6.6 md/dl in women and > 7.0 mg/dl in men) were associated with coronary heart disease in women only, and this persisted after adjustment for hypertension and nephropathy^23^. In the other, the Steno Diabetes Center study (n = 676), higher SU concentrations were associated with CVD on univariate analysis, but the association was not independent of traditional CVD risk factors^24^.

### Longitudinal associations between baseline urate levels and macrovascular disease

We noted sex differences in the relationship of SU to subsequent MACE (associated in women only), but not to “any CVD” (no association in either sex). The definition of “any CVD” encompasses a wider range of clinical scenarios than MACE, including revascularization procedures (Table 2), and may represent a less accurate criterion for defining macrovascular disease. Moreover, the relatively low number of event components limited statistical power. We demonstrated that in women, SU as a continuous variable remained significantly associated with MACE after adjustment for age, mean DCCT/EDIC HbA1c, triglycerides, and MetS, but not after adjustment for albuminuria, a risk factor for macrovascular disease and for elevated SU concentrations; however, the association remained significant if the final adjustment was for eGFR rather than albuminuria. Loss of significance after adjustment for microalbuminuria suggests that the association of SU with MACE in women is mediated by renal dysfunction.

Quartiles of SU were not significantly associated with subsequent “any CVD” or MACE in either sex. A more rigorous stratified analysis of SU in both sexes, which did not assume equal vascular event risk in men and women, demonstrated borderline statistical significance. In concert with these findings, other studies in the general population have noted sex disparities, with hyperuricemia predicting risk for CVD and mortality risk more effectively in women than men^7,25–27^, even though SU levels are usually lower in women.

Other T1D studies, from the Steno Diabetes Centre and the Preventing Early Renal Loss in Diabetes (PERL) trial that related serum urate to future hard clinical events are smaller, and/or have fewer events and/or shorter follow-up time than our DCCT-EDIC study^28,29^. Also, they do not present sex-specific analyses but instead include sex as a variable in a cohort-wide analysis, if at all. In the Steno Diabetes Center study (n = 670) with median follow-up 5.2 and 6.2 years for cardiovascular events and mortality, respectively, higher baseline urate concentrations were associated with significantly higher risk of cardiovascular events, mortality and renal decline, even after adjustment for multiple risk factors^28^. Unlike the Steno Study, we reported results for each sex separately. However, using a more rigorous sex-stratified analysis, which did not assume similar event risk for men and women, and after adjustment for multiple covariates, our results did not reach statistical significance.

The PERL trial (n = 530) used an entirely different approach, testing the effect of reducing SU concentrations with allopurinol. After ~ 3 years follow-up, there was no effect on the rate of decline in GFR (primary end-point) or the incidence of CVD events (secondary end-point)^29^.

With regard to associations of SU with surrogate measures of macrovascular disease in T1D, the Coronary Artery Calcification in Type 1 Diabetes (CACTI) Study found that SU predicted CAC progression independent of conventional CVD risk factors (including MetS) in those without renal disease^30^. In another CACTI cohort analysis, baseline SU predicted CAC progression even after adjusting for many traditional risk factors, and addition of baseline SU concentrations to the model improved the related C-statistic and non-event reclassification index^31^. Sex-specific analyses were not presented^31^. Our study, which is not concordant, provides sex-specific analyses, uses ‘hard’ cardiovascular events rather than surrogate end-points, and has longer follow-up.

### Strengths and limitations

Our study’s strengths include its focus on T1D, similar numbers of men and women, and especially its use of the well-characterized DCCT/EDIC cohort with long follow-up and hard clinical end-points. Limitations include the low numbers of subjects with elevated serum urate concentrations, low macrovascular event rates, large number of potential covariates, use of a single SU measure, lack of a non-diabetic comparator group, lack of detailed documentation of exposure to drugs that alter SU levels (e.g. thiazides, lipid drugs, allopurinol), and lack of genetic data. SU was measured several years after the randomization phase (DCCT) was completed; however, our analyses are adjusted for mean updated HbA1c minimizing the risk of bias in this regard. More data will be available in future as vascular events accrue in the DCCT/EDIC.

## Conclusions

In the well-characterized DCCT/EDIC cohort, we found generally normal serum urate concentrations that were higher in men than women. SU was strongly associated with features of the MetS and CRP in both sexes. Over a median of 14-years follow-up, baseline SU was associated with MACE in women only, but this association did not persist in a full multivariate analysis. Our study does not support the routine measurement of urate in early-middle aged people with T1D as a means to assess cardiovascular risk. Further studies in the DCCT/EDIC cohort will evaluate longer periods of follow-up as age advances and cardiovascular events accrue.

## Data Availability

Data collected for the DCCT/EDIC study through June 30th 2017 are available to the public through the NIDDK Repository (https://repository.niddk.nih.gov/studies/edic/). Data collected in the current cycle (July 2017–June 2022) will be available within 2 years after the end of the funding cycle.
